# Supplementary material for: Pharmacological Inhibition of SLC33A1 Promotes Endoplasmic Reticulum Hyperoxidation and Induces Adaptive IRE1/XBP1s Signaling
Source: bioRxiv. 2026 Feb 18:2026.02.17.706344. Preprint. [Version 1] doi: 10.64898/2026.02.17.706344 (PMC12934820; doi:10.64898/2026.02.17.706344)
Supplement: Supplement 5 [file NIHPP2026.02.17.706344v1-supplement-5.pdf]

bioRxiv preprint doi: https://doi.org/10.64898/2026.02.17.706344; this version posted February 18, 2026. The copyright holder for this preprint (which was not certified by peer review) is the author/funder, who has granted bioRxiv a license to display the preprint in perpetuity. It is made available under aCC-BY 4.0 International license.

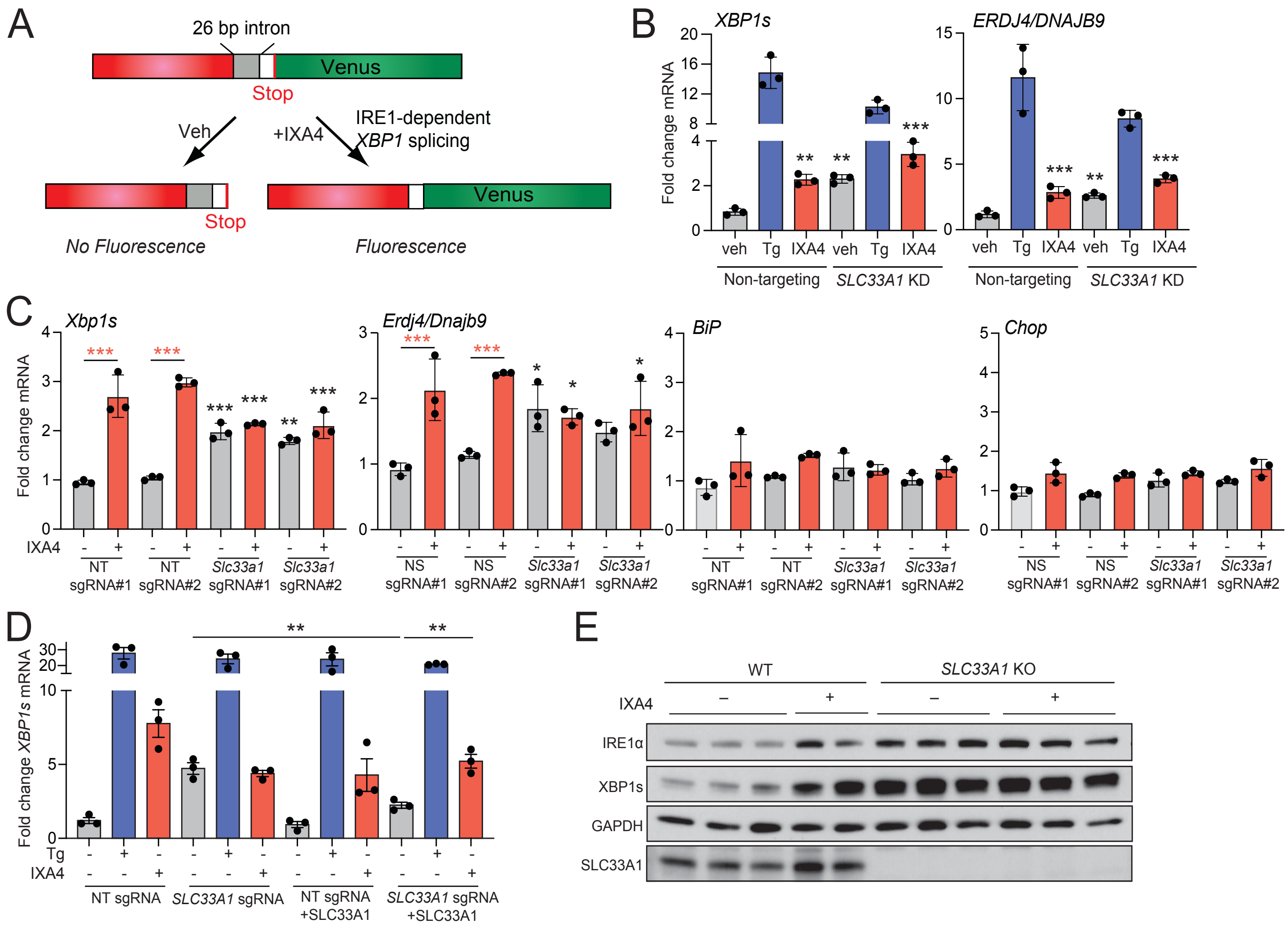

Figure S1 (Related to Figure 1). CRISPR screen identifies SLC33A1 as a protein involved in IXA4-induced IRE1/XBP1s signaling. A. Schematic of the XBP1-Venus IRE1 splicing reporter. **B**. Expression, measured by qPCR, of the IRE1/XBP1s target genes *XBP1s* and *ERDJ4/DNAJB9* in HEK293 cells expressing non-targeting or *SLC33A1* shRNA and treated for 4 h with Tg (0.5 μM) or IXA4 (10 μM). **C**. Expression, measured by qPCR, of the IRE1 target genes *Xbp1s* and *Erdj4/Dnajb9*, the ATF6 target gene *BiP*, and the PERK target gene *Chop* in MEF cells CRISPR-deleted of *Slc33a1* using two distinct sgRNA and treated for 4 h with IXA4 (10 μM). MEF cells expressing two distinct non-targeting sgRNAs are shown as a control. **D**. Expression, measured by qPCR, of the IRE1 target gene *XBP1s* in non-targeting HEK293 cells or HEK293 cells lacking *SLC33A1* transfected with mock or wild-type SLC33A1, as indicated, and then treated for 4 h with Tg (0.5 μM) or IXA4 (10 μM). **E**. Immunoblots of the indicated proteins in HEK293T cells expressing non-targeting or *SLC33A1* sgRNAs treated for 4 h with IXA4 (10 μM). Black asterisks in (**C**) represent comparisons between vehicle-treated control cells expressing NT sgRNA #1 and mutant cells treated with or without IXA4. Red asterisks in (**C**) indicate comparisons between vehicle and IXA4-treated samples. *p<0.05, **p<0.01, ***p<0.005, one-way ANOVA.

bioRxiv preprint doi: https://doi.org/10.64898/2026.02.17.706344; this version posted February 18, 2026. The copyright holder for this preprint (which was not certified by peer review) is the author/funder, who has granted bioRxiv a license to display the preprint in perpetuity. It is made available under aCC-BY 4.0 International license.

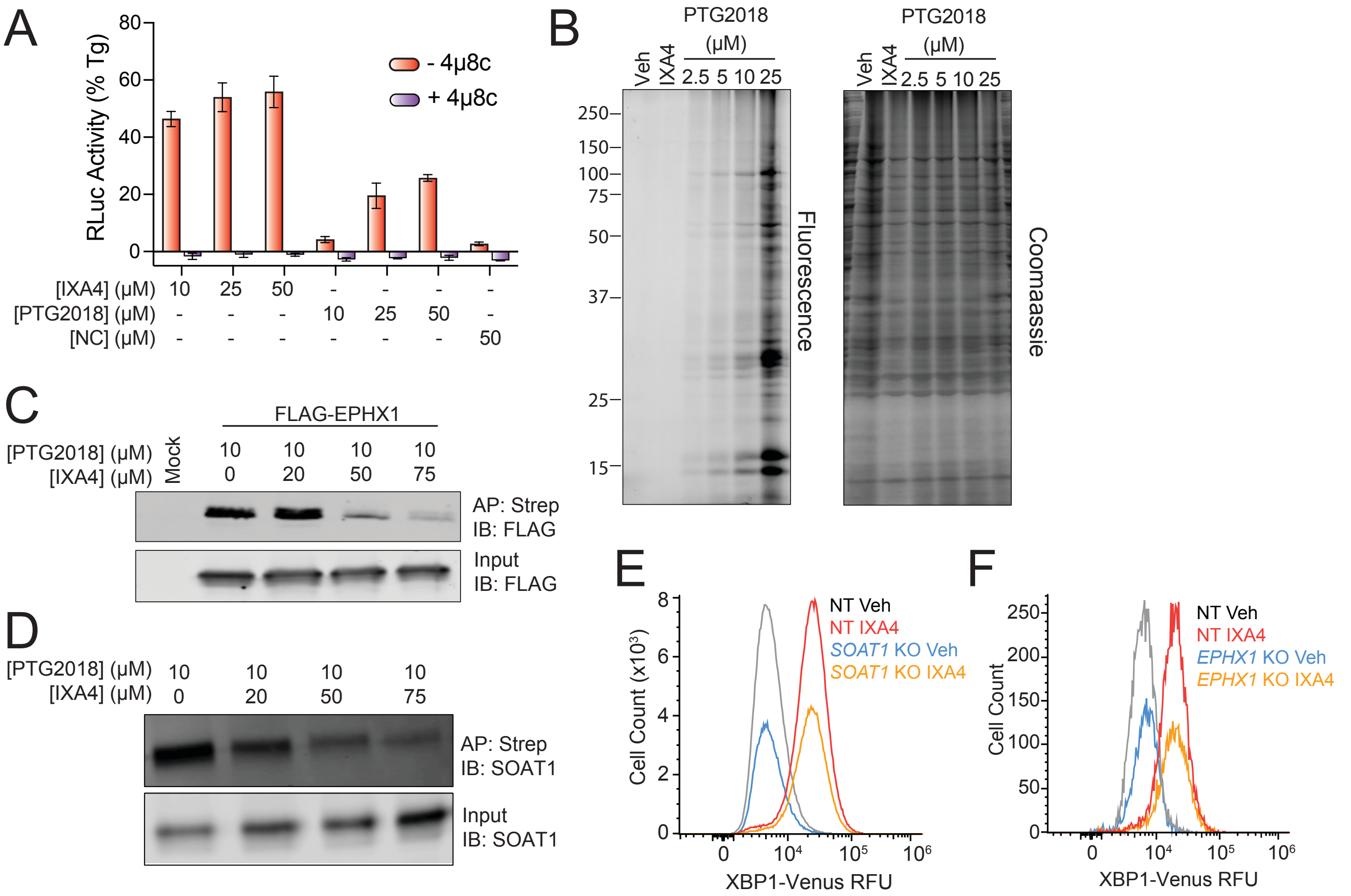

Figure S2 (Related to Figure 2). IXA4 binds SLC33A1. **A.** XBP1-Rluc signal in HEK293 cells stably expressing XBP1-RLuc treated with the indicated concentration of IXA4 or PTG2018 in the presence or absence of the IRE1 RNAse inhibitor 4μ8c (32 μM) for 14 h. Data are normalized to the XBP1-RLuc signal seen in cells treated with thapsigargin (Tg; 0.5 μM). Error bars show SEM for n=3 replicates. **B**. Dose-dependent proteome labeling with PTG2018 (left, rhodamine fluorescence; right, Coomassie stain). PTG2018-labeled proteins were identified by appending TAMRA-azide to PTG2018 via click chemistry. **C**. Streptavidin affinity purification and proteomes (input) from HEK293T cells overexpressing FLAG-tagged EPHX1 and treated with the indicated concentration of PTG2018 and IXA4. PTG2018 labeling of FLAG-tagged EPHX1 was visualized by appending a biotin to the alkyne handle of PTG2018 using click chemistry and monitoring the recovery of FLAG-tagged protein in streptavidin isolates. Mock transfected cells are shown as a control. **D**. Streptavidin affinity purification and proteomes (input) from HEK293T cells treated with the indicated concentration of PTG2018 and IXA4. PTG2018 labeling of SOAT1 was visualized by appending biotin to the alkyne handle of PTG2018 via click chemistry and monitoring the recovery the recovery of endogenous SOAT1 in streptavidin isolates. **E**, **F**. XBP1-Venus signal, measured by flow cytometry, in HEK293 cell stably expressing Cas9 and transduced with lentivirus encoding non-targeting (NT), *SOAT1 or EPHX1* sgRNA treated for 14 h with vehicle or IXA4 (10 μM).

bioRxiv preprint doi: https://doi.org/10.64898/2026.02.17.706344; this version posted February 18, 2026. The copyright holder for this preprint (which was not certified by peer review) is the author/funder, who has granted bioRxiv a license to display the preprint in perpetuity. It is made available under aCC-BY 4.0 International license.

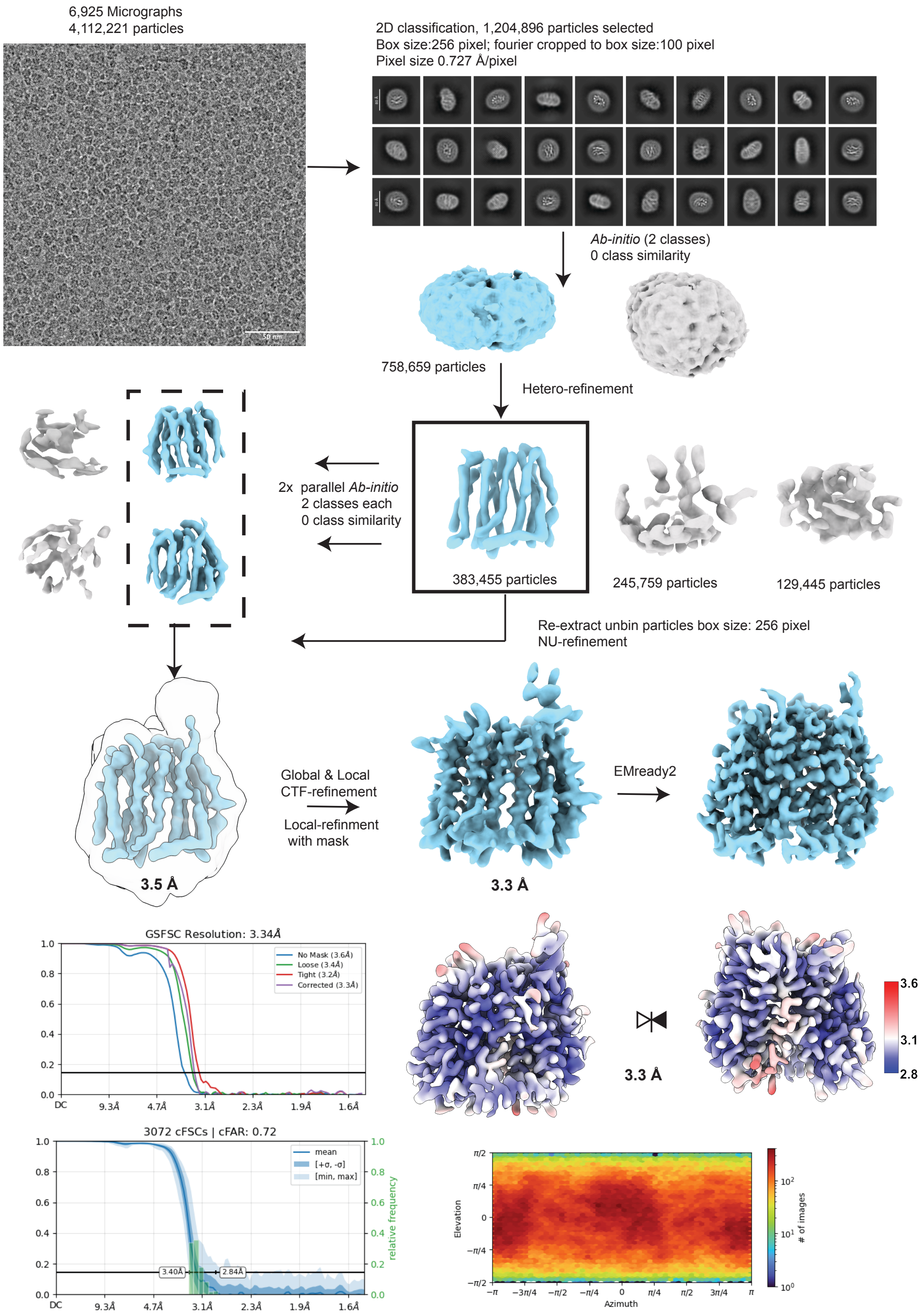

Figure S3 (Related to Figure 3). Cryo-EM image-processing workflows for apo SLC33A1. Image-processing workflow for apo SLC33A1. Representative micrographs with 5Å applied lowpass and 2D class averages are shown. Selected particles from good classes were used to generate ab initio reconstructions (two classes) and subsequently refined by heterogeneous refinement to isolate a well-resolved class. In parallel, two additional ab initio reconstructions (two classes each) were performed, and the best-resolved volume was used as the reference for downstream refinement. Particles from the well-resolved heterogeneous-refinement class were then re-extracted to full box size and refined by non-uniform refinement, followed by global and local CTF refinement and masked local refinement. The final reconstruction was post-processed with EMReady2, and validation outputs including gold-standard FSC, cFSC, angular distribution, and local-resolution estimation in Å are shown.

bioRxiv preprint doi: https://doi.org/10.64898/2026.02.17.706344; this version posted February 18, 2026. The copyright holder for this preprint (which was not certified by peer review) is the author/funder, who has granted bioRxiv a license to display the preprint in perpetuity. It is made available under aCC-BY 4.0 International license.

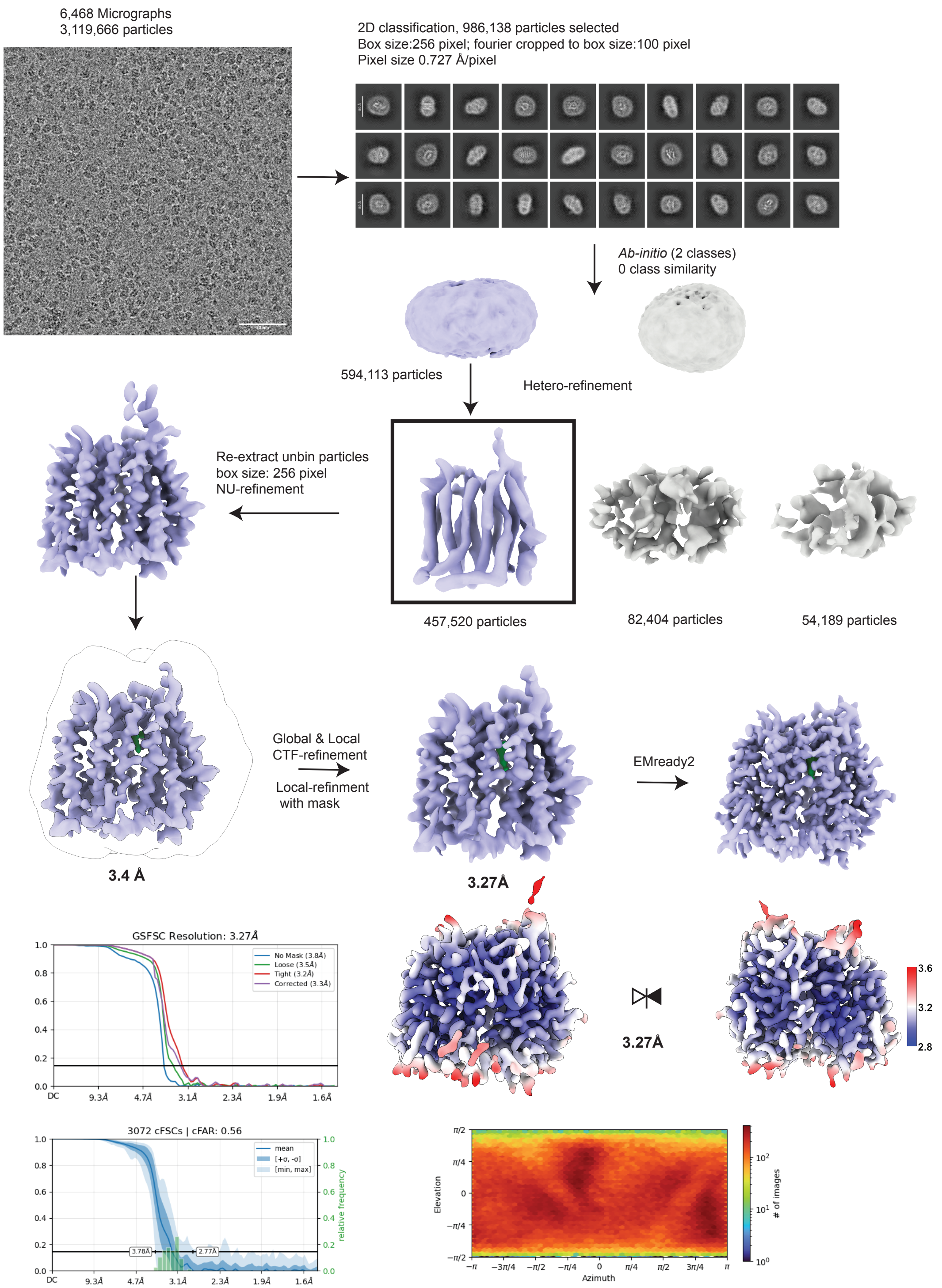

Figure S4 (Related to Figure 3). Cryo-EM image-processing workflows for IXA4-bound SLC33A1. Image-processing workflow for IXA4-bound SLC33A1. Representative micrographs with 5Å applied lowpass and 2D class averages are shown. Particles from selected 2D class averages were used to generate ab initio models, which served as references for heterogeneous refinement. Particles from the well-resolved class were re-extracted and refined by non-uniform refinement, followed by global and local CTF refinement and masked local refinement. The final map was post-processed with EMReady2. Corresponding gold-standard FSC, cFSC, angular distribution, and local-resolution estimation in Å are shown.

bioRxiv preprint doi: https://doi.org/10.64898/2026.02.17.706344; this version posted February 18, 2026. The copyright holder for this preprint (which was not certified by peer review) is the author/funder, who has granted bioRxiv a license to display the preprint in perpetuity. It is made available under aCC-BY 4.0 International license.

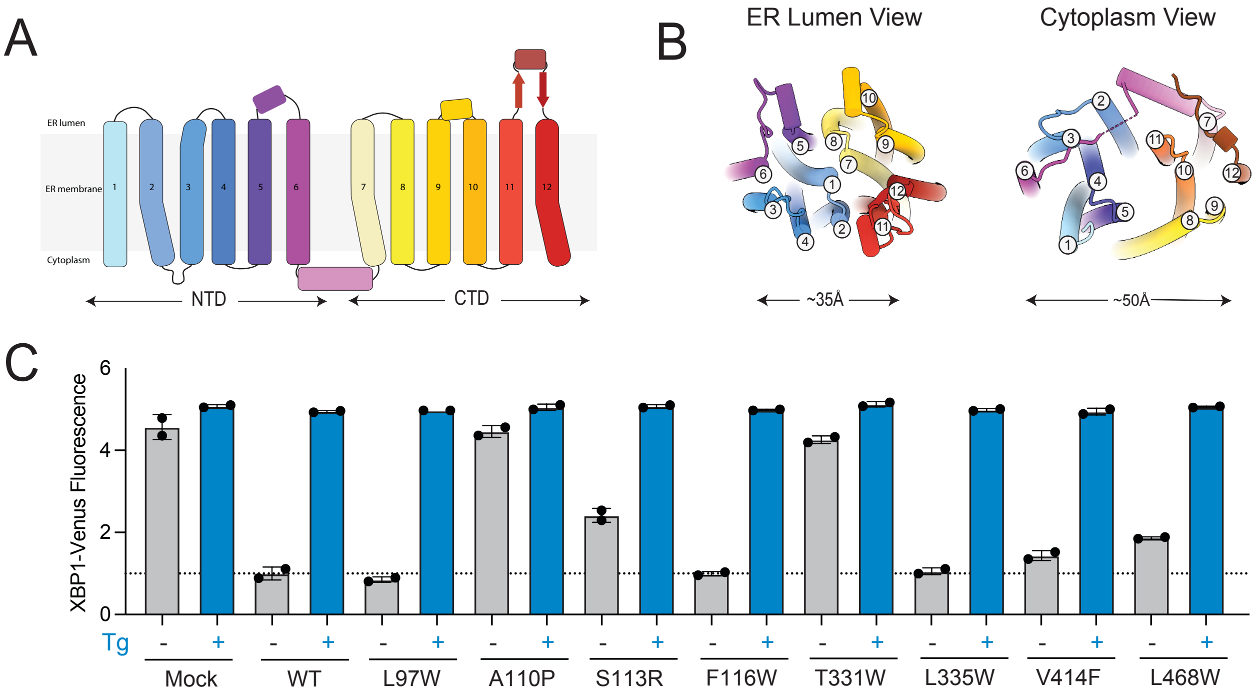

Figure S5 (Related to Figure 3). IXA4 binds the central channel of SLC33A1. **A**. Topology diagram of SLC33A1 showing 12 transmembrane helices (TM1-TM12) and their orientation in the ER membrane. TM helices are colored from N to C terminus as light blue, cornflower blue, dodger blue, steel blue, violet, magenta, lemon chiffon, yellow, gold, orange, tomato, and firebrick. The lateral helix is colored plum, and the terminal segment is colored saddle brown. **B.** Ribbon representations of SLC33A1 in the ER-lumen view (left) and the cytoplasmic view (right), illustrating the closed and open conformations, respectively. Transmembrane helices are colored and numbered as in panel **A**. **C.** XBP1-Venus signal, measured by flow cytometry, after reconstitution of wild-type SLC33A1 and mutant proteins in HEK293 XBP1-Venus reporter cells lacking endogenous SLC33A1 following treatment with vehicle or Tg (0.5 μM) for 14 h.

bioRxiv preprint doi: https://doi.org/10.64898/2026.02.17.706344; this version posted February 18, 2026. The copyright holder for this preprint (which was not certified by peer review) is the author/funder, who has granted bioRxiv a license to display the preprint in perpetuity. It is made available under aCC-BY 4.0 International license.

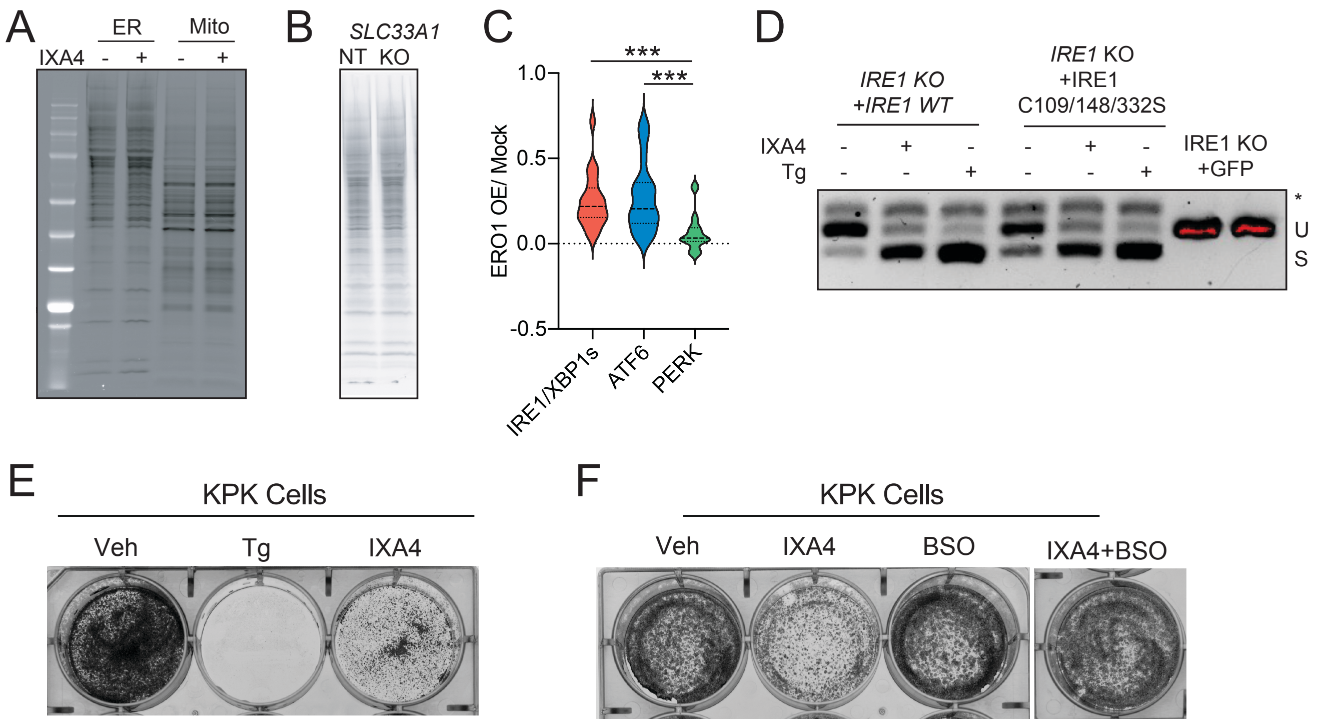

Figure S6 (Related to Figure 4). IXA4 binding to SLC33A1 promotes ER hyperoxidation and IRE1 activation A. In-gel fluorescence of acetylated proteins in ER and mitochondrial fractions isolated from HEK293T cells treated for 3 h with IXA4 (10 μM) and then incubated for 1 h with 3-butynoic acid. Acetylated proteins were identified by appending a rhodamine tag onto the 3-butynoic acid probe via click chemistry. **B**. In-gel fluorescence of acetylated proteins in ER fractions isolated from HEK293T Cas9 cells expressing non-targeting (NT) or *SLC33A1* sgRNA incubated for 1 h with the 3-butynoic acid probe. Acetylated proteins were then identified by appending a rhodamine tag onto the 3-butynoic acid probe via click chemistry. **C**. Expression (published DNA microarrays)[38] of IRE1/XBP1s, ATF6, and PERK target genesets[48] in HEK293T cells overexpressing the ER oxidase ERO1. Source data available in GEO (GSE40601). **D**. *XBP1* mRNA splicing (RT-PCR) in *IRE1*-deficient HeLa cells transfected with wild-type IRE1 or a C109S/C148S/C332S IRE1 triple mutant and treated for 2 h with IXA4 (10 μM) or thapsigargin (Tg; 0.5 μM). Unspliced (u), spliced (s), and hybrid (*), *XBP1* are shown. **E**. Crystal violet stained KPK cells treated for 2 days with thapsigargin (Tg; 0.5 μM) or IXA4 (10 μM). **F**. Crystal violet stained KPK cells incubated for 2 days with IXA4 (10 μM) and/or BSO (50 μM). p<0.05, **p<0.01, ***p<0.005, one-way ANOVA.

bioRxiv preprint doi: https://doi.org/10.64898/2026.02.17.706344; this version posted February 18, 2026. The copyright holder for this preprint (which was not certified by peer review) is the author/funder, who has granted bioRxiv a license to display the preprint in perpetuity. It is made available under aCC-BY 4.0 International license.

SUPPLEMENTAL TABLE LEGENDS

Table S1 (related to Figure 1). CRISPR screen identifies SLC33A1 as a protein involved in IXA4-induced IRE1/XBP1s signaling
Phenotypes, fold change and pvalue (Table S1A) and counts (Table S1B) for genes targeted in the CRISPR screen for DMSO and IXA4-treated cells were analyzed using MAGeCK (see Methods for details).

Table S2 (related to Figure 2). IXA4 binds SLC33A1
Fold change and pvalue with a corresponding competition excess (Table S2A) and TMT data (Table S2B) for proteins identified a competition chemoproteomics experiment for IXA4 and PTG2018-treated cells

Table S3 (related to Figure 3). IXA4 binds the central channel of SLC33A1
Cryo-EM data collection, refinement, and validation

Table S4 (additional materials). Table showing the primers used to for qPCR and RT-qPCR, and protospacers used for CRISPR in this manuscript.
